# Supplementary material for: A Novel Biosensor and Algorithm to Predict Vitamin D Status by Measuring Skin Impedance
Source: Sensors (Basel). 2021 Dec 4;21(23):8118. doi: 10.3390/s21238118 (PMC8662433; doi:10.3390/s21238118)
Supplement: Supplementary file 1 [file sensors-21-08118-s001.zip › sensors-1481915-supplementary.pdf]

Supplementary Data S1

Table S1. Participant data characteristics (InBody)

Variable	Unit	Frequency analysis/Descriptive statistics (N=26)
**Basic Information**		
Age	yr	28.73 ± 9.28
Gender		male: 14(53.8%); female: 12(46.2%)
**Obesity analysis**		
Body mass index	Kg/m^2	21.84 ± 2.14
Body fat percentage	%	25.55 ± 8.05
**Body composition analysis**		
Body water	L	34.07 ± 7.3
Protein	Kg	9.18 ± 2.05
Minerals	Kg	3.23 ± 0.61
Body fat	Kg	15.59 ± 4.72
Muscle mass	Kg	43.79 ± 9.46
Lean body mass	Kg	46.47 ± 9.94
**Skeletal muscle/Fat analysis**		
Skeletal muscle mass	Kg	25.7 ± 6.14
**Muscle analysis by region**		
Right arm	Kg	2.37 ± 0.73
Right arm	%	90.06 ± 9.17
Left arm	Kg	2.34 ± 0.74
Left arm	%	89.04 ± 9.67
Body	Kg	20.68 ± 4.46
Body	%	95.19 ± 5.37
Right leg	Kg	7.34 ± 1.65
Right leg	%	96.84 ± 6.95
Left leg	Kg	7.34 ± 1.69
Left leg	%	96.59 ± 6.74
**Analysis of extracellular water ratio**		
Extracellular water ratio		0.38 ± 0.01
**Body fat analysis by region**		
Right arm	Kg	0.97 ± 0.4
Left arm	Kg	0.99 ± 0.41
Body	Kg	7.73 ± 2.68
Right leg	Kg	2.43 ± 0.64
Left leg	Kg	2.42 ± 0.64
**Other items**		
Intracellular water	L	21.24 ± 4.7
Extracellular water	L	12.83 ± 2.61
Basal metabolic rate	Kcal	1373.81 ± 214.73
Abdominal fat percentage		0.87 ± 0.06
Body cell mass	Kg	30.42 ± 6.72

Supplementary Data S2

Table S2. Characteristics of participant's health checkup data

Variable	Frequency analysis/Descriptive statistics (N=26)	Variable	Frequency analysis/Descriptive statistics (N=26)
**Basic Information**		**Sugar test**	
Age	28.73 ± 9.28	Blood sugar (before meals)	89.04 ± 10.01
Gender	male: 14(53.8%); female: 12(46.2%)	HbA1c-NGSP	5.24 ± 0.43
Blood type (Rh+)	A: 7(26.9%); AB: 2(7.7%); B: 8(30.8%); O: 9(34.6%)	yolk	Negative: 25(96.2%)
**Body measurements**		Insulin, fasting 1 time	9.56 ± 3.67
Waist circumference	80.03 ± 7.31	HOMA Ratio	2.06 ± 0.92
Body mass index	21.48 ± 2.17	**kidney/Pancreas/Other**	
**Blood pressure**		BUN	12.42 ± 2.67
Blood pressure (highest)	119.73 ± 13.59	Uric Acid	5.41 ± 1.12
Blood pressure (lowest)	73.46 ± 10.13	Na	138.81 ± 2.05
Pulse	76.92 ± 9.63	K	4.59 ± 1.06
**Eye examination**		P	3.79 ± 0.46
Corrected visual acuity (left)	1.09 ± 0.25	Creatinine	0.78 ± 0.18
Corrected visual acuity (right)	1.06 ± 0.25	Amylase	67.58 ± 21.05
Naked eyesight (left)	0.91 ± 0.44	Ca	9.53 ± 0.25
Naked eyesight (right)	0.96 ± 0.43	CKD-EPI	115.75 ± 11.24
Intraocular pressure (left)	15.32 ± 2.94	MDRD	108.13 ± 17.21
Intraocular pressure (right)	15.84 ± 2.87	**Tumor marker test (female)**	
Fundus examination	Normal Limit: 20(76.9%)	AFP(EIA)	1.72 ± 0.61
**hearing test**		CEA	0.98 ± 0.58
Airway Hearing 500Hz (Left)	11.73 ± 5.47	CA19-9	15.36 ± 28.13
Airway Hearing 500Hz (Right)	10.38 ± 1.96	CA-125	15.86 ± 5.78
Airway Hearing 1000Hz (Left)	11.35 ± 4.14	HE4	29.23 ± 4.73
Airway Hearing 1000Hz (Right)	11.15 ± 4.96	ROMA, premenopausal	2.28 ± 0.86
Airway Hearing 2000Hz (Left)	12.12 ± 6.19	ROMA, postmenopausal	7.13 ± 2.4
Airway Hearing	11.65 ± 6.32	**Tumor marker test (male)**	

2000Hz (Right)			
Airway Hearing 4000Hz (Left)	14.04 ± 11.23	AFP(EIA)	2.32 ± 1.35
Airway Hearing 4000Hz (Right)	15.58 ± 16.57	CEA	2 ± 1.1
Airway Hearing 8000Hz (Left)	16.73 ± 16.79	CA19-9	8.69 ± 4.93
Airway Hearing 8000Hz (Right)	16.54 ± 14.88	PSA	1.07 ± 0.84
**lung function test**		**hepatitis test**	
FVC	4.1 ± 0.86	HBs-Ag	Negative: 26(100%)
FVC(%)	90.5 ± 10.66	HBs-Ab	Negative: 13(50%); Positive: 13(50%)
FEV1	3.47 ± 0.75	Anti-HBc(Total)	Negative: 26(100%)
FEV1(%)	95.92 ± 10.73	Anti-HCV(EIA)	Negative: 26(100%)
PEF	7.33 ± 1.89	Anti-HAV(Total)	Negative: 15(57.7%); Positive: 11(42.3%)
FEV1/FCV	85.69 ± 7.57	**immune serum test**	
PEF(%)	90.57 ± 16.69	AIDS	Negative: 26(100%)
**general blood test**		TPHA	Negative: 26(100%)
HB	14.63 ± 1.44	Rheumatoid factor (quantitative)	<10: 26(100%)
HCT	42.96 ± 3.82	C-reactive protein	0.1 ± 0.03
WBC	6.12 ± 1.46	Syphilis test (qualitative)	Negative: 26(100%)
RBC	4.8 ± 0.43		
Platelet	280.65 ± 54.84	**thyroid**	
MCV	89.57 ± 2.63	Free T4	1.41 ± 0.21
MCH	30.47 ± 1.23	TSH	2.21 ± 1.42
MCHC	34.03 ± 0.92	Thyroid hormone T3	1.09 ± 0.19
RDW	12.06 ± 0.41	**Stool test**	
PDW	11.12 ± 1.27	Protozoa	Negative: 18 (69.2%)
ESR	10.69 ± 7.78	Worm	Negative: 18(69.2%)
MPV	9.95 ± 0.74	Stool occult blood test	5.06 ± 12.14
**leukocyte**		Whipworm	-
Neutrophil	54.5 ± 6.63	Fluke	Negative: 18(69.2%)
Lymphocyte	33.91 ± 5.73	**urinalysis**	
Mono	8.13 ± 1.61	Yolk	Negative: 24(92.3%)
Eoso	2.85 ± 1.78	Protein	Negative: 15(57.7%); Weakly positive: 7(26.9%)
Baso	0.61 ± 0.27	Occult Blood	Negative: 23(88.5%); Weakly positive: 1(3.8%)
**Lipid test**		pH	5.61 ± 0.5
Total cholesterol	178.31 ± 39.16	LEUKOCYTE	Negative: 23(88.5%); Positive: 1(3.8%)

HDL cholesterol	63.54 ± 13.3	Uro-Bilinogen	Negative: 22(84.6%); Weakly positive: 2(7.7%)
LDL cholesterol	92.04 ± 32.3	Bilirubin	Negative: 20(76.9%); Positive: 4(15.4%)
TG	103.04 ± 48.58	Nitrite	Negative: 24(92.3%)
**liver function test**		Ketone	Negative: 17(65.4%); Weakly positive: 5(19.2%); Positive: 2(7.7%)
AST	18.88 ± 6.02	S.G	1.03 ± 0.005
ALT	17.77 ± 10.47	Urine color	Amber: 12(46.2%); Brown: 11(42.3%); Pale Yellow: 1(3.8%)
r-GPT	23.5 ± 17.35	Bacteria	Negative: 15(57.7%); A few: 5(19.2%); Moderate: 3(11.5%); Many: 1(3.8%)
Albumin	4.78 ± 0.17	**Urine sediment**	
Total Protein	7.29 ± 0.28	RBC	0~2: 24(92.3%)
Total Bilirubin	0.7 ± 0.38	WBC	0~2: 21(80.8%); 3~5: 2(7.7%); 11~20: 1(3.8%)
Direct Bilirubin	0.26 ± 0.11	Epithelial cells	0~2: 16(61.5%); 3~5: 5(19.2%); 6~10: 3(11.5%)
ALP	57.46 ± 23.06	**Other blood tests**	
LDH	361.23 ± 58.29	Vitamin D	30.03 ± 13.14
A/G ratio	1.93 ± 0.27		

Supplementary Data S3

Table S3. Variables considered after normality test (InBody)

variable	Unit	descriptive statistics (N=26)	Significance Probability (*p*-value)
**Obesity analysis**			
Body mass index	Kg/m^2	21.84 ± 2.14	.658
Body fat percentage	%	25.55 ± 8.05	.083
**Body composition analysis**			
Minerals	Kg	3.23 ± 0.61	.315
Body fat	Kg	15.59 ± 4.72	.615
Lean body mass	Kg	46.47 ± 9.94	.053
**Muscle analysis by region**			
Right arm	%	90.06 ± 9.17	.446
Left arm	%	89.04 ± 9.67	.895
Log_ torso	%	4.55 ± 0.06	.057
Log_right leg	%	4.57 ± 0.07	.070
Left leg	%	96.59 ± 6.74	.116
**Analysis of extracellular Water ratio**			
Extracellular water secretion		0.38 ± 0.01	.923
**Body fat analysis by region**			
Right arm	Kg	0.97 ± 0.4	.470
Left arm	Kg	0.99 ± 0.41	.262
Body	Kg	7.73 ± 2.68	.917
Right leg	Kg	2.43 ± 0.64	.401
Left leg	Kg	2.42 ± 0.64	.356
**Other items**			
Extracellular water	L	12.83 ± 2.61	.067
Basal metabolic rate	Kcal	1373.81 ± 214.73	.051
Abdominal fat percentage		0.87 ± 0.06	.220
